# Correlation between Genotypic and Phenotypic Testing for Resistance to Rifampin in *Mycobacterium tuberculosis* Clinical Isolates in Haiti: Investigation of Cases with Discrepant Susceptibility Results

**DOI:** 10.1371/journal.pone.0090569

**Published:** 2014-03-05

**Authors:** Oksana Ocheretina, Vincent E. Escuyer, Marie-Marcelle Mabou, Gertrude Royal-Mardi, Sean Collins, Stalz C. Vilbrun, Jean W. Pape, Daniel W. Fitzgerald

**Affiliations:** 1 Center for Global Health, Division of Infectious Diseases, Department of Medicine, Weill Cornell Medical College, New York, New York, United States of America; 2 Les Centres GHESKIO, Port-au-Prince, Haiti; 3 Laboratory of Mycobacteriology, Wadsworth Center, New York State Department of Health, Albany, New York, United States of America; St. Petersburg Pasteur Institute, Russian Federation

## Abstract

The World Health Organization has recommended use of molecular-based tests MTBDRplus and GeneXpert MTB/RIF to diagnose multidrug-resistant tuberculosis in developing and high-burden countries. Both tests are based on detection of mutations in the Rifampin (RIF) Resistance-Determining Region of DNA-dependent RNA Polymerase gene (*rpoB*). Such mutations are found in 95–98% of *Mycobacterium tuberculosis* strains determined to be RIF-resistant by the “gold standard” culture-based drug susceptibility testing (DST).

We report the phenotypic and genotypic characterization of 153 consecutive clinical *Mycobacterium tuberculosis* strains diagnosed as RIF-resistant by molecular tests in our laboratory in Port-au-Prince, Haiti. 133 isolates (86.9%) were resistant to both RIF and Isoniazid and 4 isolates (2.6%) were RIF mono-resistant in MGIT SIRE liquid culture-based DST. However the remaining 16 isolates (10.5%) tested RIF-sensitive by the assay.

Five strains with discordant genotypic and phenotypic susceptibility results had RIF minimal inhibitory concentration (MIC) close to the cut-off value of 1 µg/ml used in phenotypic susceptibility assays and were confirmed as resistant by DST on solid media. Nine strains had sub-critical RIF MICs ranging from 0.063 to 0.5 µg/ml. Finally two strains were pan-susceptible and harbored a silent *rpoB* mutation.

Our data indicate that not only detection of the presence but also identification of the nature of *rpoB* mutation is needed to accurately diagnose resistance to RIF in *Mycobacterium tuberculosis*. Observed clinical significance of low-level resistance to RIF supports the re-evaluation of the present critical concentration of the drug used in culture-based DST assays.

## Introduction

The World Health Organization (WHO) recommends using rapid molecular tests MTBDRplus and GeneXpert MTB/RIF to diagnose tuberculosis (TB) and multi-drug resistant tuberculosis (MDR-TB) in developing and high-burden countries [1], [2]. Molecular tests dramatically shorten time to diagnosis from months to days (MTBDRplus) or hours (GeneXpert MTB/RIF). Both tests are based on PCR amplification of the beta subunit of mycobacterial DNA-dependent RNA Polymerase (*rpoB*) followed by detection of mutations in its 81 bp Rifampin Resistance-Determining Region (RRDR). Such mutations are found in 95-98% of all *Mycobacterium tuberculosis* (MTB) strains resistant to Rifampin (RIF) [3], [4]. Furthermore, most isolates resistant to RIF are also resistant to Isoniazid (INH) therefore the presence of *rpoB* mutations can be used as a surrogate marker for MDR-TB [5], [6].

Haiti is representative of countries where the TB burden is high but resources and laboratory facilities are very limited. It has the highest rate of TB in the Western Hemisphere, with an estimated prevalence of 331 per 100 000 population [7]. In 2008 2.9% of newly diagnosed TB cases were MDR-TB [8]. The TB laboratory of the Groupe Haïtien d'Etude du Sarcome de Kaposi et des Infections Opportunistes (GHESKIO) in the capital Port-au-Prince is the only laboratory in a country with population of over 10 millions with the capacity to perform mycobacterial culture and Drug Susceptibility Testing (DST) and serves as a national reference facility.

In 2008, the GHESKIO TB laboratory introduced rapid molecular PCR-based tests for pre-screening of primary specimens and MTB isolates for resistance to RIF to make better use of the limited capacity for mycobacterial culture and culture-based DST and to shorten time to MDR-TB diagnosis. While recent publications consistently report high sensitivity of such tests in clinical samples, there are conflicting reports about the extent of their specificity with a range from 100% [9] to as low as 57% [10].

Here we present examination of 153 consecutive clinical MTB strains diagnosed as resistant to Rifampin with molecular tests in our laboratory between March 2008 and July 2012. Sixteen of them (10.5%) demonstrated discrepant RIF susceptibility results between molecular and “gold standard” culture-based susceptibility tests.

## Materials and Methods

### 1. Ethics Statement

Institutional review boards at Weill Cornell Medical College and GHESKIO Centres approved the study of MTB clinical isolates with a limited amount of unlinked clinical data. The IRBs waived the need for written informed consent from the participants.

### 2. Study Site

GHESKIO is the largest HIV and TB treatment center in Haiti. GHESKIO counsels and HIV tests 30,000 patients annually, provides ART to 6,000 HIV infected patients, and treats over 1,000 patients annually for TB. GHESKIO's BSL3 level TB laboratory serves as a national reference facility. The laboratory is certified by the Division of AIDS, US National Institute of Health through the annual laboratory audit and successful participation in required EQA programs. The results in EQA programs for Mycobacteriology (College of American Pathologists); GeneXpert MTB/RIF (2 programs - CDC and TBGX Monitor of DAIDS Clinical Trials Group) and LPA assays (WHO Stop TB Department) were 100% successful since 2008.

### 3. Molecular testing

#### 3.a. GenoType MTBDRplus

In 2008 the GHESKIO TB laboratory introduced GenoType MTBDRplus (Hain Life Sciences, Nehren, Germany) PCR-based assay for rapid detection of resistance to RIF. MTBDRplus was used to test decontaminated AFB smear-positive samples and MTB isolates where direct testing was not performed or did not generate results. Testing was performed according to the manufacturer's recommendations.

#### 3.b. GeneXpert MTB/RIF

In May 2011 GeneXpert MTB/RIF (Cepheid, CA, USA) real-time PCR test became accessible and was introduced in the laboratory to replace MTBDRplus for direct testing of clinical specimens irrespective of their AFB status. While GenoType MTBDRplus was performed on DNA extracts, GeneXpert MTB/RIF test was performed directly on clinical samples without prior extraction according to the manufacturer's instructions. To analyze culture isolates with GeneXpert MTB/RIF, 100 µl of bacterial suspension from a positive MGIT tube were mixed with 2 ml of the sample reagent supplied with the kit and tested the same way as recommended for clinical samples.

#### 3.c. Sanger Sequencing

DNA was extracted from culture isolates as described previously [11] and analyzed with PCR and sequencing for the presence of mutations in 7 genes linked to resistance to RIF (*rpoB*), INH (*katG, inhA, aphC*), EMB (*embB*), PZA (*pncA*) and fluoroquinolones (*gyrA*). Primers, PCR conditions and analyzed fragments are outlined in Table 1.

**Table 1:** Primers used for sequencing and analyzed fragments.

Locus	Primer	Sequence (5′ - 3′)	Annealing T°	Amplicon (analyzed fragment) size, bp	Flanking sequences of analyzed fragment
*rpoB*	rpoB_F	ccacccaggacgtggaggcgatcacac	55°	359 (81)	[ggcacc…gcgctg]
	rpoB_R	cgtttcgatgaacccgaacgggttgac			
*katG*	katG_F	gaaacagcggcgctgatcgt	60°	527 (30)	[gacgcg…gtcgta]
	katG_R	gagttgaatgactcctggatc			
*inhA-reg*	inhA-reg_F	cctcgctgcccagaaaggga	60°	248 (130)	[cgttac…gaggaa]
	inhA-reg_R	atcccccggtttcctccggt			
*oxyR-aphC*	aphC_F	cgcaacgtcgactggctcata	60°	359 (217)	[cttggc…gcgact]
	aphC_R	gcctgggtgttcgtcactggt			
*embB*	embB_F	tgatattcggcttcctgctc	55°	417 (343)	[catcct…cctat]
	embB_R	accgctcgatcagcacatag			
*pncA*	pncA_F	gctggtcatgttcgcgatcg	58°	720 (581)	[cccgaa…tcctga]
	pncA_R	tgcttgcggcgagcgctcca			
*gyrA*	gyrA_F	cagctacatcgactatgcga	60°	320 (232)	[aagccc…cggcgg]
	gyrA_R	gggcttcggtgtacctcatc			

#### 3.d. Spoligotyping

Spoligotyping was performed at the New York State Department of Health (NYSDOH) Mycobacteriology Laboratory by using the standard membrane-based method [12]. Patterns were assigned a Spoligo International Type (SIT) number according to the SITWITWEB International database (http://www.pasteur-guadeloupe.fr:8081/SITVIT_ONLINE/).

### 4. Processing of specimens and mycobacterial culture

Clinical specimens were decontaminated using NaOH/NALC method as recommended by the United States Center for Disease Control and Prevention [13]. The laboratory used commercially available media for all mycobacterial culture (Becton Dickenson, Franklin Lakes, NJ, USA). Each specimen was cultured on solid (Lowenstein-Jensen slant) and liquid (BACTEC MGIT 960 tube) media. Capilia TB-Neo (Tauns Laboratories Inc., Izunokuni, Japan) or SD-Bioline (Standard Diagnostics Inc., Yongin, Korea) rapid tests for detection of Ag MPT64 were used to identify MTB isolates.

### 5. Phenotypic drug susceptibility testing

All isolates carrying *rpoB* mutations underwent drug susceptibility testing (DST) for first-line anti-tuberculosis drugs in Haiti with BACTEC MGIT 960 SIRE and PZA kits according to the manufacturer's instructions. Drugs were tested at a concentration of 1.0 µg/mL for STM, 0.1 µg/mL for INH, 1.0 µg/mL for RIF, 5.0 µg/mL for EMB and 100 µg/mL for PZA.

The same isolates were sent to NYSDOH Mycobacteriology Laboratory where BACTEC MGIT 960 DST was repeated. Additionally susceptibility testing to INH, RIF, EMB, STM, rifabutin, capreomycin, cycloserine, ethionamide (ETA), kanamycin, amikacin, p-aminosalicylc acid (PAS), and ofloxacin (OFL) was performed with the proportion method on 7H10 medium agar as recommended by the Clinical and Laboratory Standards Institute [14].

Three to 5 days old positive MGIT cultures were used to determine Minimal Inhibitory Concentration (MIC) to RIF in a microplate assay [15]. MGIT tubes were vortexed vigorously for 2 min to break up clumps and let stand for 10 min. 100 µl of the bacterial suspension were added to the wells of a 96-well plate containing serial two-fold dilutions of RIF (8–0.031 µg/ml) in 100 µl of 7H9 Middlebrook medium. Outer wells on the plate perimeter were filled with water to prevent medium evaporation. For each isolate three control wells without drug were set up – first with medium only, second with 100 µl of bacterial suspension and third with 100 µl of 1∶100 dilution of bacterial suspension in 7H9 Middlebrook medium. Each isolate was tested in triplicate. After incubation at 37°C for 5 days, 20 µl of Alamar Blue and 12 µl of 10% Tween-80 (Fisher Scientific, Pittsburgh, PA, USA) solution in sterile water were added to the control well containing the 1∶100 bacterial dilution and the plate was returned to the 37°C. When the color of the 1∶100 control well changed from blue to purple/pink indicating sufficient bacterial growth, Alamar Blue and Tween-80 were added to the remaining wells and MIC readings were performed after 24-hour incubation in 37°C. A control *M. tuberculosis* strain ATCC25177 was included with every MIC experiment and each batch of DST on 7H10 agar. Same Rifampin (Sigma Aldrich, Catalog #R3501) was used for both assays.

## Results

### 1. Molecular testing for resistance to Rifampin

From March 2008 to June 2012 primary specimens and MTB isolates from 4352 patients were screened with molecular methods in our laboratory in Haiti and 162 of those patients were diagnosed as resistant to RIF (Table S1). Two cases detected with MTBDRplus, and 7 cases detected with GeneXpert MTB/RIF directly in primary specimens did not produce cultures. MTB was isolated in the remaining 153 cases. Depending on which method was used initially to detect RIF resistance, the 153 isolates were re-tested retrospectively so that ultimately each isolate was examined by both MTBDRplus and GeneXpert MTB/RIF. Concordance between the two molecular tests was 100%.

### 2. Genotypic and phenotypic analysis of 153 strains harboring rpoB mutations

DNA sequencing identified *rpoB* mutations in all 153 RIF-resistant isolates previously detected with MRTBDRplus and GeneXpert MTB/RIF (Table 2). The most common genotype S531L was found in 53.6% of all samples. Four *rpoB* mutations included on the MTBDRplus test strip – S531L, D516V, H526Y and H526D – accounted for 75% of the cases. Additionally, eighteen different *rpoB* genotypes (9 single missense mutations, 3 double missense mutations, 1 silent mutation, 1 codon insertion, and 4 deletions of 1, 2, 3 or 4 codons) were found among the remaining 25%.

**Table 2:** Sequencing, culture-based DST and Spoligotyping of 153 isolates resistant to RIF as determined by GeneXpert MTB/RIF and Hain MTBDRplus assays.

			Resistance to 1 µg/ml Rifampin*		Spoligotypes of isolates (SIT)
rpoB mutations	n of strains	% of 153	MGIT SIRE kit	Agar proportion	
S531L	82	53.6%	**RES**	**RES**	20 different types
D516V	16	10.5%	**RES**	**RES**	5, 2, 20, 50, 1712
H526D	10	6.5%	**RES**	**RES**	2, 17, 47, 50, 93
H526Y	7	4.6%	**RES**	**RES**	2, 20, 53, 93, 2281
S531W	6	3.9%	**RES**	**RES**	7, 20, 42, 53
H526L	5	3.3%	4 S+1 **RES**	**RES**	4, 34, 93
L511P	5	3.3%	S	S	53
S531Q	5	3.3%	**RES**	**RES**	51, 53
L511P & M515T	2	1.3%	S	S	53
T508A	2	1.3%	S	S	20
T508T (silent)	2	1.3%	S	S	50
D516V & F514L	1	0.7%	**RES**	**RES**	53
F inserted after Q513	1	0.7%	**RES**	**RES**	93
H526C	1	0.7%	S	**RES**	1321
H526R	1	0.7%	**RES**	**RES**	51
L511P & D516C	1	0.7%	**RES**	**RES**	53
Q513K	1	0.7%	**RES**	**RES**	47
S222L	1	0.7%	**RES**	**RES**	51
Δ D516	1	0.7%	**RES**	**RES**	5
Δ Q517,N518	1	0.7%	**RES**	**RES**	193
Δ F514,M515,D516	1	0.7%	**RES**	**RES**	50
Δ M515,D516,Q517,N518	1	0.7%	**RES**	**RES**	5

* **RES** – Resistant.

S – Sensitive.

All 153 isolates were tested for resistance to 1 µg/ml RIF with two methods – automated MGIT 960 SIRE test in liquid media (GHESKIO Laboratory in Haiti and NYSDOH Laboratory in US) and proportion method on 7H10 agar (NYSDOH Laboratory).

When examined by MGIT SIRE DST test, 133 isolates (86.9% of 153 strains resistant to RIF by molecular tests) were found to be MDR-TB – i.e. resistant to at least RIF and INH.

Four isolates (2.6% of 153 strains resistant to RIF by molecular tests) with mutations S531L or S531W were found to be RIF mono-resistant - i.e. susceptible to other first- and second-line anti-tuberculosis drugs with the exception of Rifabutin. These 4 isolates each had a distinct spoligotype and likely were not related. Of note, three of the four patients with RIF mono-resistance were HIV infected and presented to GHESKIO after failing Category I TB treatment.

Finally 16 isolates (10.5% of 153 strains resistant to RIF by molecular tests) were found sensitive to RIF with MGIT 960 SIRE test.

### 3. Occurrence and classification of strains with discordant results between molecular and conventional tests for susceptibility to RIF

The 16 isolates with discordant results for resistance to RIF were divided between three groups based on the value of RIF MIC and the nature of the *rpoB* mutation: Borderline RIF resistant: 5 isolates (3.3%); Low-level RIF resistant: 9 isolates (5.9%) and RIF sensitive harboring silent mutation in *rpoB*: 2 isolates (1.3%). Their clinical, genotypic and phenotypic characteristics are presented in Table 3.

**Table 3:** Clinical, genotypic and phenotypic characteristics of 16 strains with discordant results for resistance to RIF.

Patients				Mutations in target genes							Culture-based DST**								Spoligotype (SIT)
ID	Age	Sex	HIV	*rpoB*	*katG*	*inhA*	*aphC*	*embB*	*pncA*	*gyrA*	RIF MIC (µg/ml)	RIF, MGIT	RIF, AP	STM	INH	EMB	PZA	Resistance to 2nd line drugs	
**Isolates with borderline resistance to Rifampin**																			
*2010-69* *	23	M	N	H526L	S315T	-	-	-	-	A90V	[2]–[4]	S	**RES**	**RES**	**RES**	S	S	**OFL**	93
*2011-111*	35	M	N	H526L	S315T	-	-	-	G23V	A90V	[2]–[4]	S	**RES**	**RES**	**RES**	**RES**	**RES**	**OFL**	93
*2011-105* *	34	M	N	H526L	S315T	-	-	M306I	-	-	1	S	**RES**	**RES**	**RES**	**RES**	S	**ETA,PAS**	4
*2009-40* *	33	M	N	H526L	S315T	-	-	M306I	-	-	1	S	**RES**	**RES**	**RES**	**RES**	S	**ETA**	4
*2011-98* *	52	M	N	H526C	S315T	-	-	G406D	L19P	-	[0.5–1]	S	**RES**	S	**RES**	**RES**	**RES**	-	1321
**Isolates with a low-level resistance to Rifampin**																			
*2011-87* *	34	F	N	L511P,M515T	S315T	-	-	M306I	-	-	[0.25–0.5]	S	S	S	**RES**	S	S	-	53
*2011-93*	10m	M	N	L511P,M515T	S315T	-	-	M306I	-	-	[0.25–0.5]	S	S	S	**RES**	S	S	-	53
*2011-102* *	30	M	P	L511P	S315T	-	-	M306I	-	-	0.125	S	S	S	**RES**	S	S	-	53
*2011-76*	28	F	P	L511P	S315T	-	-	-	-	-	[0.125–0.25]	S	S	S	**RES**	S	S	-	53
*2010-68*	21	F	N	L511P	S315T	-	-	-	-	-	0.125	S	S	S	**RES**	S	S	-	53
*2008-26* *	18	M	N	L511P	S315T	-	-	-	-	-	n.d.	S	S	S	**RES**	S	S	-	53
*2008-18*	30	F	N	L511P	S315T	-	-	-	-	-	n.d.	S	S	S	**RES**	S	S	-	53
*2011-150*	29	M	N	T508A	-	-	-	-	-	-	0.063	S	S	S	S	S	S	-	20
*2012-151*	25	M	N	T508A	-	-	-	-	-	-	0.063	S	S	S	S	S	S	-	20
**Rifampin-sensitive isolates with silent ** ***rpoB*** ** mutation**																			
*2010-152*	25	F	P	T508T	-	-	-	-	-	-	<0.031	S	S	S	S	S	S	-	50
*2008-153*	47	F	P	T508T	-	-	-	-	-	-	<0.031	S	S	S	S	S	S	-	50

* patients with history of treatment failure.

** “AP” - Agar Proportion; “**RES**” - Resistant; “S” – Sensitive.

#### 3.a. Isolates with borderline resistance to RIF

Five isolates (3.3% of 153 strains resistant to RIF by molecular tests) were susceptible to 1 µg/ml RIF by automated BACTEC MGIT 960 SIRE test but resistant to the same concentration of drug by proportion method on 7H10 agar. For these isolates we adopted the term “borderline resistant to RIF” from Van Deun et al [16], who reported discrepant DST results for a strain harboring H526L *rpoB* mutation.

MIC for RIF ranged between 0.5 and 4 µg/ml. Four isolates carried a H526L *rpoB* mutation and one isolate had a H526C mutation. Interestingly, another isolate harboring a H526L mutation with an MIC for RIF between 2 and 4 µg/ml was found resistant to RIF by both culture-based DST methods and therefore was not included into this group.

Isolates with borderline resistance to RIF were also resistant to STM (4/5), INH (5/5), EMB (4/5), PZA (2/5), Ofloxacin (2/5), Ethionamide (2/5) and PAS (1/5). Additional DNA sequencing revealed the presence of mutations in genes known to be associated with resistance to INH (*katG*), EMB (*embB*), PZA (*pncA*) and fluoroquinolones (*gyrA*). Spoligotyping grouped the 5 isolates into 3 clusters.

Of note, 4 of the 5 patients with borderline resistance to RIF presented to GHESKIO after failing Category I and Category II TB treatments.

#### 3.b. Isolates with low-level resistance to RIF

Nine isolates (5.9% of 153 strains resistant to RIF by molecular tests) were susceptible to 1 µg/ml of RIF both in solid and in liquid culture-based DST and therefore were RIF-sensitive according to criteria accepted in clinical laboratory practice. However here we describe these strains as having a low-level resistance to RIF since their RIF MICs were above 0.031 µg/ml, a consensus concentration determined in GHESKIO laboratory by testing 10 RIF-sensitive strains without *rpoB* mutations.

Two of the isolates in this group had a T508A (Acc->Gcc) *rpoB* mutation and shared identical spoligotype (SIT 20). Both isolates were pan-susceptible to first- and second-line antibiotics in culture-based DST. MIC to RIF was 0.063 µg/ml – one dilution step higher than the lowest tested RIF concentration of 0.031 µg/ml. DNA sequencing did not reveal additional mutations in other genes associated with drug-resistance. Both patients had no history of prior TB treatment and were cured with a Category I regimen.

Five isolates had a L511P (cTg->cCg) *rpoB* mutation and two had double mutations L511P and M515T (aTg->aCg). MTB strains with single L511P mutation had an MIC to RIF between 0.125 and 0.25 µg/ml. Combination of L511P and M515T mutations resulted in RIF MIC between 0.25 and 0.5 µg/ml. All 7 isolates harboring L511P mutation also had a katG mutation S315T conferring resistance to INH and shared the same spoligotype (SIT 53).

Of note, 3 of the 7 patients harboring the L511P mutation presented to GHESKIO after failing Category I and Category II TB treatment (Table 3).

#### 3.c. Isolates with silent mutation or synonymous SNP

Two specimens from independent patients identified as RIF-resistant by molecular testing in 2008 and in 2010 exhibited an identical silent mutation or synonymous single nucleotide polymorphism (sSNP) in *rpoB* codon T508 (acC->acT). Both isolates were susceptible to all tested first- and second-line anti-tuberculosis drugs as determined by culture-based DST. In MIC experiment they were susceptible to the lowest tested RIF concentration of 0.031 µg/ml. DNA sequencing revealed the absence of mutations in *katG, inhA, aphC, embB, gyrA* and *pncA* genes. The 2 isolates had the same spoligotype (SIT 50). Neither of these patients presented with prior history of TB treatment and both were cured with a Category I regimen.

## Discussion

Molecular tests MTBDRplus and GeneXpert MTB/RIF are increasingly used in developing and high-burden countries to diagnose MDR-TB, while conventional culture-based DST is considered to be a “gold standard” for MTB drug resistance testing.

89,5% of MTB isolates initially found RIF-resistant by molecular tests in our laboratory in Haiti between March 2008 and July 2012 were confirmed to be resistant to RIF by MGIT SIRE assay, the method recommended by the WHO for automated culture-based DST [5]. However the remaining 10.5% (16 cases) initially diagnosed as resistant to RIF by molecular methods tested susceptible to RIF with MGIT SIRE. This situation triggered an investigation with multiple repeated tests and posed a significant burden to our laboratory. It also undermined the confidence of clinicians in the results of molecular susceptibility tests and presented a challenge for clinical management of the 16 patients concerned.

Thorough characterization of the 16 cases uncovered a wide spectrum of resistance profiles. On one side there were patients resistant to as many as 5-6 anti-tuberculosis drugs who had already failed treatment with Category I and Category II TB regiments. On another side we found patients infected with pan-susceptible MTB strains harboring silent *rpoB* mutations. Remaining patients harbored MTB strains with a RIF MIC below the cut-off value of 1 µg/ml and with various susceptibility patterns to TB drugs other than RIF.

Discrepant results were not due to performance issues of a particular molecular method reported previously [17]–[21]. Both MTBDRplus and GeneXpert MTB/RIF yielded identical results for the 16 discordant samples and the presence of *rpoB* mutations in these isolates was confirmed by DNA sequencing. Rather they were explained by the well-known fact that not every genotypic modification of *rpoB* gene affects phenotypic resistance to RIF equally; a number of published studies describe that the value of the RIF MIC strongly correlates with the position and nature of the amino-acid substitution in *rpoB* RRDR [22], [23].

Discordant results in 5 out of 16 cases were explained by MIC values close to the critical concentration of 1 µg/ml used to define resistance to RIF. *rpoB* mutations H526L and H526C associated with such “borderline” resistance have been previously described, and DST on solid media was recommended as a preferred method to test for RIF resistance with such strains [16], [24]. However liquid culture-based MGIT DST kits are recommended in clinical practice since they provide results in 10-14 days as compared to 3-4 weeks for the agar proportion method [5], [25]. “Borderline” resistance to RIF has been strongly associated with treatment failure in our experience and in the literature [26]. For isolates with “borderline” RIF resistance, discrepant results are a consequence of the technical shortcomings of conventional DST methods with molecular tests being more sensitive and reproducible diagnostic tools.

A broad spectrum of RIF MIC values (0.063 µg/ml to 0.5 µg/ml) was observed in 9 other discrepant cases. Strains with low MICs for RIF have been historically overlooked by culture-based DST. As a consequence, there is little data about their prevalence and clinical significance [16]. Among the cases described here, 3 out of the 7 patients with low-level resistance to RIF and resistance to INH, presented to GHESKIO after failing Category I and Category II TB treatment.

Finally two of 16 discrepant cases were incorrectly identified as RIF-resistant by molecular testing because they contained silent mutation (sSNP) in *rpoB* codon T508. Silent mutations do not result in structural changes in the DNA-dependent RNA Polymerase and so do not interfere with its inhibition by RIF. Findings of silent mutations in *rpoB* RRDR is not surprising as SNPs occur every 3 kb of MTB genome [27]. Silent mutations in RRDR of *rpoB* were also reported in clinical isolates from Korea (codons L511 and Q513) [28], China (codon T525) [29], India (codons A532 and L533) [30], New Zealand (codon Q510) [31], USA (codon F514) [32], and Spain (codon F514) [33], [34]. Synonymous SNPs are likely to be selectively neutral and so can persist in the population - in Spain sSNP in *rpoB* codon F514 was registered in 0.8% of 1,450 consecutive clinical samples [33]. While the nature and frequency of silent mutations in *rpoB* varies depending on geographic location, using solely molecular tools to test for resistance to RIF in countries with limited resources will, most probably result in misdiagnosis of MDR-TB and in inappropriate treatment of TB patients with second line anti-tuberculosis drugs.

The observed complexity of molecular mechanism for resistance to RIF in MTB and the occurrence of silent mutations in *rpoB* RRBR warrant the referral of all RIF-resistant cases diagnosed by molecular methods such as MTBDRplus and GeneXpert MTB/RIF to MDR-TB treatment centers with access to a reference TB laboratory performing conventional DST and DNA sequencing. Determining the nature of *rpoB* mutations and the associated level of resistance to RIF (RIF MIC) will deepen our knowledge about molecular mechanisms of drug resistance in TB and will improve result interpretation of existing molecular-based tests. It is necessary to create a centralized user-friendly online resource for MTB drug-resistant mutations, similar to the HIV drug resistance mutations site on www.iasusa.org. Health care workers need to have an easy access to the online reference resource and be educated to understand basic principles of molecular assays' performance. This report should by no means be considered as an attempt to disparage MTBDRplus and GeneXpert MTB/RIF molecular susceptibility tests which have been already validated in multiple studies [17], [19], [35], [36]. We rather see the proposed steps as a necessary but still unfinished part of adopting the new technology to test for drug resistance in TB.

Our clinical observations raise the question about the present definition of resistance to RIF, which has been in use since 1963. MTB strains are called RIF-resistant if >1% of bacteria grows in Middlebrook medium supplemented with a critical concentration of the drug, currently set at 1 µg/ml. Critical concentration is determined as the lowest concentration to inhibit ≥95% of the bacterial growth in culture [13]. A modern approach to determine susceptibility breakpoints utilizes pharmacokinetic/pharmacodynamics modeling of MIC data generated from a large number (>1000) of clinical strains and reflects the ability of the drug to kill bacteria at the site of infection as opposed to the exclusively *in vitro* approach used 50 years ago [37], [38]. Recent studies question the clinical relevance of the current RIF breakpoint value and call for adjusting the critical concentration used to define resistance to RIF to as low as 0.0625 µg/ml [39]. Applying a 0.0625 µg/ml susceptibility breakpoint to the isolates of the present study would change susceptibility status of at least 7 of 16 discrepant cases to “resistant” and so increase the specificity of molecular tests for RIF susceptibility from 89.5% to 94.1%. Use of molecular tests rather than culture-based DST to screen large number of MTB isolates for resistance to RIF will allow detection of previously occult cases with low-level resistance and generate necessary clinical data to support evidence from pharmacokinetic studies.

To conclude, we report results of a systematic real-time genotypic and phenotypic investigation of all strains found to be resistant to RIF by molecular tests in setting of high-burden country with moderate prevalence of MDR-TB. In 10.5% of TB cases, genotypic resistance to RIF was not confirmed by phenotypic DST. Our clinical observations suggest that not only detection of the presence but also identification of the nature of *rpoB* mutation is needed for accurate diagnosis of resistance to Rifampin.

## Supporting Information

Table S1 **162 cases resistant to RIF by molecular tests -** ***rpoB*** **mutations, specimen type and availability of direct testing of clinical specimens with MTBDRplus and GeneXpert MTB/RIF.**
(XLSX)
